# Biochemical Characterisation of Phage Pseudomurein Endoisopeptidases PeiW and PeiP Using Synthetic Peptides

**DOI:** 10.1155/2015/828693

**Published:** 2015-09-21

**Authors:** Linley R. Schofield, Amy K. Beattie, Catherine M. Tootill, Debjit Dey, Ron S. Ronimus

**Affiliations:** AgResearch Limited, Grasslands Research Centre, Tennent Drive, Private Bag 11008, Palmerston North 4442, New Zealand

## Abstract

Pseudomurein endoisopeptidases cause lysis of the cell walls of methanogens by cleaving the isopeptide bond Ala-*ε*-Lys in the peptide chain of pseudomurein. PeiW and PeiP are two thermostable pseudomurein endoisopeptidases encoded by phage ΨM100 of *Methanothermobacter wolfei* and phages ΨM1 and ΨM2 of *Methanothermobacter marburgensis*, respectively. A continuous assay using synthetic peptide substrates was developed and used in the biochemical characterisation of recombinant PeiW and PeiP. The advantages of these synthetic peptide substrates over natural substrates are sensitivity, high purity, and characterisation and the fact that they are more easily obtained than natural substrates. In the presence of a reducing agent, purified PeiW and PeiP each showed similar activity under aerobic and anaerobic conditions. Both enzymes required a divalent metal for activity and showed greater thermostability in the presence of Ca^2+^. PeiW and PeiP involve a cysteine residue in catalysis and have a monomeric native conformation. The kinetic parameters, *K*_*M*_ and *k*_cat_, were determined, and the *ε*-isopeptide bond between alanine and lysine was confirmed as the bond lysed by these enzymes in pseudomurein. The new assay may have wider applications for the general study of peptidases and the identification of specific methanogens susceptible to lysis by specific pseudomurein endoisopeptidases.

## 1. Introduction

**Figure 1:**
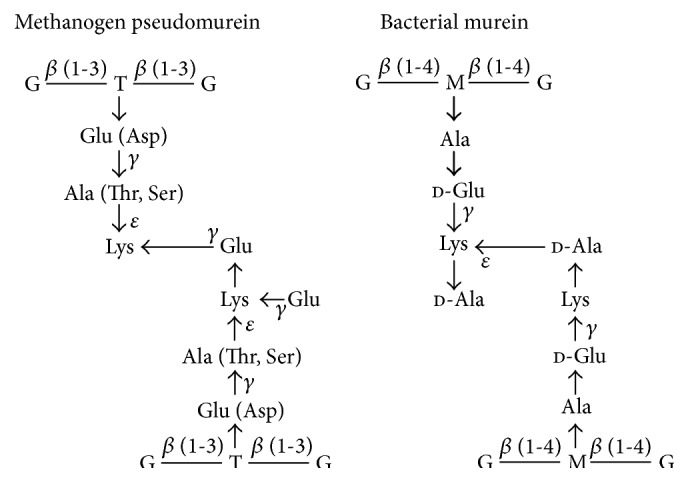
Comparison of the composition of pseudomurein and murein from cell walls. G,* N*-acetylglucosamine; T,* N*-acetyltalosaminuronic acid; M,* N*-acetylmuramic acid. Methanogen pseudomurein contains l-amino acids, *β* (1-3) bonds, and* N*-acetyltalosaminuronic acid in the glycan chain and several unusual isopeptide bonds. Glu can be replaced by Asp; Ala can be replaced by Thr or Ser, in some strains. Bacterial murein contains d- and l-amino acids and *β* (1-4) bonds and* N*-acetylmuramic acid in the glycan chain. The figure was adapted and modified from Hartmann and König [5] and Kandler and König [3].

Pseudomurein is one of the many different cell wall polymers present in Archaea, being found only in Methanobacteriales and the genus* Methanopyrus* [1–4]. Pseudomurein has a similar overall 3-dimensional structure to murein, the predominant component of bacterial cell walls. The main chemical differences in the methanogen pseudomurein cell walls (Figure 1) are (i) the presence of an archaeal-specific sugar,* N*-acetyltalosaminuronic acid in the glycan backbone (instead of the bacterial* N*-acetylmuramic acid) that is linked to either* N*-acetylglucosamine or* N*-acetylgalactosamine (rather than just* N*-acetylglucosamine), (ii) the use of *β* (1-3) bonds in the glycan chain, instead of *β* (1-4) bonds, (iii) the lack of d-amino acids in the peptide chain (instead of a mixture of d-, l-, and* meso*-amino acids), and (iv) the use of *ε*- and *γ*-isopeptide bonds in both the peptide and interpeptidyl cross link [3, 5–7]. These differences result in the pseudomurein-containing methanogens being resistant to lysozyme and other bacterial cell wall hydrolases.

Pseudomurein endoisopeptidases (Pei) cause lysis of the cell walls of methanogens by cleaving the isopeptide bond Ala-*ε*-Lys in the peptide chain in pseudomurein [8, 9]. PeiW and PeiP are two thermostable pseudomurein endoisopeptidases found in members of the order Methanobacteriales [10, 11]. PeiW is encoded by the defective prophage ΨM100 of the thermophile* Methanothermobacter wolfei* [12], and PeiP is encoded by phage ΨM1 and deletion mutant ΨM2 of the thermophile* Methanothermobacter marburgensis* [13, 14]. Other phages characterised from the Methanobacteriales include prophage Φmru from* Methanobrevibacter ruminantium* [15] and ΦF1 and ΦF3 which are associated with* Methanothermobacter* species [16]. Φmru from* Mbb. ruminantium* encodes a lytic enzyme, endoisopeptidase PeiR, which was shown to lyse* Mbb. ruminantium* cells in pure culture but shows little homology to PeiW and PeiP [15].

Phage endoisopeptidases have helped to overcome the impermeable nature of the methanogen cell wall. They have been used in a fluorescence* in situ* hybridisation (FISH) procedure [17], for the gentle removal of cell walls in the isolation of high molecular weight and undegraded DNA and in the preparation of protoplasts of pseudomurein-containing methanogens [8].

PeiW and PeiP share only about 53% amino acid identity [9] but both cleave isopeptide bonds formed between the *ε*-amino group of an l-Lys residue and the *α*-carboxyl group of an l-Ala residue in the peptide chain in pseudomurein [14]. PeiW and PeiP share the same catalytic triad of conserved cysteine, histidine, and aspartate residues as transglutaminases and thiol proteases. Transglutaminases catalyse the formation of *γ*-Glu-*ε*-Lys cross links between proteins, while PeiW and PeiP cleave the similar Ala-*ε*-Lys bond. Interestingly, a *γ*-Glu-*ε*-Lys bond is thought to be the major cross-linking bond for the formation of pseudomurein [7].

The catalytic endoisopeptidase domain of PeiW and PeiP is at the C-terminus of each enzyme. The N-terminus consists of a pseudomurein cell wall binding domain containing four pseudomurein binding motifs [10, 18]. The PeiW pseudomurein cell wall binding domain has been shown to be responsible for binding to pseudomurein, which facilitates endopeptidase activity [18–21].

PeiW has been shown to cleave the Ala-*ε*-Lys bond of* Methanothermobacter thermautotrophicus* pseudomurein [8]. In addition, PeiW, or a recombinantly produced enzyme, has also been used to lyse* Mtb. marburgensis*,* Mtb. thermautotrophicus*,* Methanobacterium bryantii*,* Methanobrevibacter arboriphilus*,* Methanosphaera stadtmanae*, and* Mbb. ruminantium* [9, 17, 22, 23]. PeiP has been shown to lyse* Mtb. marburgensis* [9, 13].

PeiW and PeiP have been partially characterised biochemically [9, 13]; however, there are few methods available to assay PeiW and PeiP endoisopeptidase activity. The most common assays use natural substrates: living methanogen cultures [14, 23], harvested methanogen cells resuspended in solution [8, 9, 13, 23], or purified methanogen cell walls in suspension [23]. Bush [24] has used harvested methanogen cells in an agarose plate and, additionally, casein, BSA, and their azo-derivatives to assay lytic enzymes from other sources. However, methanogen cells and cell walls are not ideal as substrates as they can be variable and are difficult and time consuming to produce and the assays are often anaerobic, discontinuous, and nonlinear [8]. Hence, there are limitations in their use for enzyme characterisation, including kinetic studies. An alternative is synthetic substrates, which are sensitive, are well characterised, have high purity, and are more easily obtained and the activity can be continuously measured spectrophotometrically. While no synthetic substrates have previously been used with PeiW or PeiP, synthetic peptides have been tested against lytic enzymes from other sources [24, 25]. In order to develop a more convenient and reproducible method for characterising pseudomurein endoisopeptidases and to explore their substrate specificities, we have developed a series of synthetic peptides that mirror the bonds within the peptide chain of pseudomurein. This work describes the development of a continuous synthetic peptide assay which could be used at elevated temperatures and the expression, purification, and biochemical characterisation of recombinant PeiW and PeiP.

## 2. Materials and Methods

### 2.1. General Methods

Electrophoresis in the presence of SDS was performed by the method of Laemmli [26] with a 4% (w/v) stacking gel and a 12% (w/v) running gel, using a Mini Protean III cell (Bio-Rad, USA). Samples were prepared in a standard loading buffer containing SDS and boiled for 5 min before application. Low range SDS-PAGE molecular weight standards (Bio-Rad, USA) were used. After electrophoresis, gels were stained using Coomassie Brilliant Blue R 250. Protein concentrations were determined by the method of Bradford [27], using bovine serum albumin as a standard. Spectrophotometric measurements were performed using a He*λ*ios *γ* UV-Vis Spectrophotometer (Thermo Scientific, USA). The pH of buffers was adjusted at room temperature. All pH values are reported as at the temperature of use and allow for Δp*K*_*a*_/°C. When required, metal ions were removed from buffer and reagent solutions by treatment with Chelex 100 resin (Bio-Rad, USA). Solutions were then filtered through a 0.45 *μ*m membrane prior to use.

*Mtb. marburgensis* phage ΨM2 (DSM 12792) was obtained from DSMZ, Germany. Methanogen cultures from our culture collection,* Mtb. wolfei* (DSM 2970),* Methanobrevibacter gottschalkii* (DSM 11977),* Methanobrevibacter smithii* (DSM 861),* Mbb. ruminantium* strain 31A (Genbank HM624055),* Msph. stadtmanae* (DSM 3091),* Methanobacterium formicicum* strain BRM9 (Genbank X99138),* Mtb. thermautotrophicus* ΔH (DSM 1053),* Methanobrevibacter* sp. SM9 (Genbank AJ009958),* Mbb. ruminantium* strain M1 (DSM 1093),* Methanospirillum hungatei* (DSM 864), and* Methanosarcina barkeri* strain CM1 (Genbank AJ002476), were grown anaerobically at 39°C essentially as described by Leahy et al. [15]. The cells were harvested (12,000 g, 15 min, 4°C), washed with 50 mM Mops pH 7.0 containing 1 mM DTT, 7.3 mM K_2_HPO_4_, and 5% glycerol (v/v), and resuspended at 50 mg mL^−1^ in the same buffer before storing at −20°C until further use. These cell suspensions were used for cell suspension and plate lysate assays.

### 2.2. Enzyme Substrates

Synthetic peptides,* p*-nitroaniline (pNA), l-Ala-pNA (ApNA), and l-Glu-*γ*-pNA (E*γ*pNA), were obtained from Sigma-Aldrich (New Zealand), H-Glu-Ala-pNA (EApNA) was obtained from Bachem AG (Switzerland), and Glu-*γ*-Ala-pNA (E*γ*ApNA), Glu-*γ*-Ser-pNA (E*γ*SpNA), Glu-*γ*-Thr-pNA (E*γ*TpNA), and Asp-*β*-Ala-pNA (D*β*ApNA) were obtained from JPT Peptide Technologies (Germany).

### 2.3. Cloning

Primers were designed using the annotated gene sequence of PeiW (Genbank accession number AAG39976) and PeiP (AAC27067). The primers PeiWfor 5′-CACCATGGAAGTGGGGCTAAATG-3′ and PeiWrev 5′-TTATCACTTGTCATAAACAGGACC-3′ were used for the amplification of the PeiW gene from* Mtb. wolfei* (DSM 2970) genomic DNA, and the primers PeiPfor 5′-ATGAGATCTAATAGTGTGAATATTG-3′ and PeiPrev 5′-TCATTAGTCATGTTTGGGGGAGAC-3′ were used for the amplification of the PeiP gene from* Mtb. marburgensis* phage ΨM2 DNA (DSM 12792), by PCR using high-fidelity AccuPrime polymerase (Invitrogen). A double stop codon was inserted in both reverse primers to prevent reading through. The amplified fragments were each cloned into the expression vector pET151D (Invitrogen) and the DNA sequences of the clones were verified (sequencing performed by the DNA Sequencing Facility at Massey University, New Zealand). Plasmids PeiW10 or PeiP18 were used to transform competent* Escherichia coli* BL21-Rosetta 2 cells (Novagen).

### 2.4. Expression and Purification

Single colonies of* E. coli* BL21-Rosetta 2 cells containing the PeiW10 or PeiP18 plasmid were precultured for approximately 7 h in 10 mL LB medium containing 100 *μ*g mL^−1^ ampicillin and 34 *μ*g mL^−1^ chloramphenicol at 37°C. Approximately 8 mL preculture was used to inoculate 800 mL of autoinduction medium ZYP-5052 [28] containing 100 *μ*g mL^−1^ ampicillin and 34 *μ*g mL^−1^ chloramphenicol in a 2 L baffled flask. Cells were grown for approximately 16 h with vigorous shaking at 30°C before harvesting (4,100 g, 15 min, 4°C), freezing, and storage at −20°C. Cell pellets were thawed and resuspended in 4-5 volumes lysis buffer (50 mM Tris pH 7.5 containing 2 mM DTT, 300 mM NaCl, 10 mM imidazole, 1% Triton X-100 (v/v), 20% glycerol (v/v), 5 mM CaCl_2_, and 10 mM MgCl_2_). Complete EDTA-free protease inhibitor (Roche, New Zealand) was added as a stock solution following the manufacturer's instructions. Lysozyme (Sigma-Aldrich L8676, New Zealand) was added to a final concentration of 1 mg mL^−1^, followed by gentle agitation for 30–60 min at room temperature. DNase (Sigma-Aldrich D5025, New Zealand) and RNase (Sigma-Aldrich R4642, New Zealand) were added to a final concentration of 5 *μ*g mL^−1^ each, followed by gentle agitation for 30–60 min at room temperature. Cell debris was removed by centrifugation (4,100 g, 15 min, 4°C). The hexahistidine-tagged enzyme was purified from the cell-free extract using nickel-affinity chromatography. The filtered enzyme was applied (1 mL min^−1^) to a HIS-Select nickel-affinity gel (Sigma-Aldrich, New Zealand) column (2.5 × 18 cm) equilibrated with 20 mM Tris pH 7.5 containing 1 mM DTT, 0.3 M NaCl, and 20 mM imidazole. The column was washed with the equilibration buffer before fractions were eluted with a linear gradient of 20–250 mM imidazole (3 mL min^−1^). Fractions were examined by SDS-PAGE and those containing protein of the expected molecular mass were pooled and concentrated using a stirred ultrafiltration cell (Amicon) with a 10 kDa nominal molecular weight limit (NMWL) membrane. The imidazole was removed and buffer exchanged, using a Bio-Gel P-6DG (Bio-Rad) column (2.5 × 17 cm) equilibrated with 20 mM Mops pH 7.0 containing 1 mM DTT, 0.3 M NaCl, and 20% glycerol (v/v) (1 mL min^−1^). Fractions containing protein were collected and concentrated using a stirred ultrafiltration cell (Amicon) with a 10 kDa nominal molecular weight limit membrane. All chromatographic steps were performed at room temperature using a BioLogic LP system (Bio-Rad) with detection at 280 nm. Purified protein was snap-frozen in liquid nitrogen and stored at −20°C until further use.

### 2.5. Cell Suspension Assays

The decrease in optical density over time of a cell suspension of* Mtb. thermautotrophicus* was monitored at 600 nm [24]. Measurements were made aerobically using 1 cm path length stoppered quartz cuvettes in a thermostated cuvette holder. Measurements were made anaerobically using 1 cm path length anaerobic septum cap quartz cuvettes. Cuvettes were mixed thoroughly, immediately before each measurement to resuspend cells. Between measurements, cuvettes were maintained at the required temperature in a thermostated water bath. Assays were performed in duplicate.

The assay reaction mixtures contained 210 *μ*L* Mtb. thermautotrophicus* cells (up to 50 mg mL^−1^ in 50 mM Mops pH 7.0 containing 1 mM DTT, 7.3 mM K_2_HPO_4_, and 5% glycerol (v/v)), 10 mM DTT, and either 10 mM MgCl_2_ or CaCl_2_ in 50 mM Bistris propane buffer at pH 7.0 and were preincubated at 60°C for 7–10 min. The cell suspension was diluted to give an initial OD_600_ of approximately 1.0. The purified enzymes and assay components were incubated overnight at room temperature: aerobic samples on the bench, anaerobic samples in a CO_2_/H_2_ (90 : 10 v/v) atmosphere. The pH of the aerobic samples was adjusted to match that of the anaerobic samples which had altered due to the CO_2_/H_2_ atmosphere. The reaction was initiated by the addition of either 2 *μ*L of PeiW or 2 *μ*L of PeiP. The total volume in all assays was 800 *μ*L.

### 2.6. Plate Lysate Assays

The method of Bush [24] was modified slightly. A solution of 22.75 mL of 1.1% boiled agarose (Axygen Biosciences) containing 0.16 M NaCl was cooled; then, 500 *μ*L cells (50 mg mL^−1^ in 50 mM Mops pH 7.0 containing 1 mM DTT, 7.3 mM K_2_HPO_4_, and 5% glycerol (v/v)), DTT, MgCl_2_, CaCl_2_, and Mops buffer at pH 7.0 were mixed in to give final concentrations of 2 mM DTT, 1 mM MgCl_2_, 1 mM CaCl_2_, and 50 mM Mops, in a total volume of 25 mL. The mixture was poured into a 100 × 15 mm plastic Petri dish. Wells were cut into the solidified agarose with a trimmed plastic dropper (diameter 4 mm). A control plate was made omitting cells. Samples of 28–40 *μ*L of PeiW (0.2 mg), PeiP (0.2 mg), or control buffer were pipetted into the wells and assay plates were incubated in a gas tight chamber under CO_2_ for 16 h at 39°C. The diameter of the zone of clearing and/or whitening was measured to give a comparison between different methanogen cell substrates.

### 2.7. Synthetic Peptide Assays

The production of pNA was monitored at 405 nm. Measurements were made aerobically (unless otherwise stated) using 1 cm path length stoppered quartz cuvettes maintained at the required temperature in a thermostated cuvette holder. Anaerobic measurements were made using anaerobic septum cap quartz cuvettes. Initial rates of reaction were measured within two minutes and were determined by a least squares fit of the initial rate data. One unit of activity is defined as the production of 1 *μ*mol of pNA per minute at the stated temperature. The extinction coefficient of pNA at 405 nm was determined by measuring the absorbance of a known concentration of pNA under assay conditions (50 mM Bistris propane buffer at pH 7.85 containing 10 mM TCEP and 10 mM CaCl_2_ at 60°C). The extinction coefficient was found to be 10,140 M^−1^ cm^−1^.

Synthetic peptide solutions were prepared by dissolving a weighed amount of each in a measured volume of methanol or 7.5% acetic acid (v/v) and diluting with 100 mM Bistris propane pH 6.5. Metal ions were removed from buffer and reagent solutions by treatment with Chelex 100 resin (Bio-Rad). Solutions were then filtered through a 0.45 *μ*m membrane prior to use. PeiW and PeiP preparations contained 0.5 mM EDTA to sequester residual metal ions.

The standard aerobic assay reaction mixture contained either 25 *μ*L (41 *μ*g) PeiW or 12 *μ*L (50 *μ*g) PeiP, 10 mM TCEP, and 10 mM CaCl_2_ in 50 mM Bistris propane buffer at pH 7.85 and was preincubated at 60°C for 7–10 min. The reaction was initiated by the addition of 15 *μ*L E*γ*ApNA to give a final concentration of 150 *μ*M. The total volume was 800 *μ*L. The reaction mixture of anaerobic assays contained either 25 *μ*L (41 *μ*g) PeiW or 62 *μ*L (189 *μ*g) PeiP, 10 mM TCEP, and 10 mM CaCl_2_ in 50 mM Bistris propane buffer at pH 7.85 and was preincubated at 60°C for 7–10 min. The reaction was initiated by the addition of 300 *μ*L prewarmed E*γ*ApNA to give a final concentration of 150 *μ*M. The total volume was 800 *μ*L. The anaerobic assays were compared to aerobic assays that used exactly the same amount of PeiW or PeiP. The purified enzymes and assay components for anaerobic assays were incubated overnight at room temperature in a CO_2_/H_2_ (90 : 10 v/v) atmosphere, and the assay components for the comparison of aerobic samples were incubated overnight at room temperature on the bench. The pH of the aerobic samples was adjusted to match that of the anaerobic samples which had altered due to the CO_2_/H_2_ atmosphere. To check the linearity of the assay with respect to the amount of enzyme, the assay reaction mixture contained either 20–60 *μ*L (33–99 *μ*g) PeiW or 8–25 *μ*L (32–100 *μ*g) PeiP and assays were initiated by the addition of substrate to remove the effect of adding different volumes of enzyme. Assays to determine the effect of pH on activity contained 50 mM Bistris propane (pH 5.36–8.76), Ampso (pH 7.08–9.08), or Caps (3-(cyclohexylamino)-1-propanesulfonic acid) (pH 9.64–10.64). All pH values are reported as at the temperature of use. Assays to determine the effect of temperature on activity contained 50 mM Bistris propane buffer with the pH adjusted to give pH 7.85 at the temperature of use (20–90°C). Divalent metal salts used in assays to restore activity to the EDTA-treated enzyme were dissolved in water which had been pretreated with Chelex. The final concentration of divalent metal salt was 10 mM in the assay reaction mixture. All metal concentrations reported have been corrected for the presence of EDTA. The metal salts used were MgCl_2_·6H_2_O (BDH), CaCl_2_·2H_2_O (Biolab), CoCl·6H_2_O (BDH), CuSO_4_·5H_2_O (Ajax), BaCl_2_·2H_2_O (BDH), MnCl_2_·4H_2_O (BDH), and NiCl_2_·6H_2_O (BDH). We were unable to use ZnCl_2_ (BDH) and FeCl_2_·4H_2_O (Fluka) due to precipitation in the assay. Activity was also measured in the absence of divalent metal ions. Assays for the determination of kinetic parameters of PeiW contained 12–700 *μ*L E*γ*ApNA (0.114–6.64 mM) and the reaction was initiated by the addition of 4 *μ*L PeiW (6.6 *μ*g) to remove the effect of adding different volumes of substrate. Assays for the determination of kinetic parameters of PeiP contained 5–75 *μ*L E*γ*ApNA (0.0475–0.713 mM) and were initiated by the addition of 30 *μ*L PeiP (91.5 *μ*g) to remove the effect of adding different volumes of substrate. No difference in activity was found in initiating the reaction with enzyme or substrate. Kinetics assays were performed in at least duplicate. *K*_*M*_ and *k*_cat_ values were determined by fitting the data to the Michaelis-Menten equation using GraFit [29].

Inhibitors tested were E64 (dissolved in 50% ethanol (v/v)), dansylcadaverine (dissolved in DMSO), PMSF (dissolved in ethanol), cystamine (dissolved in water), and* N*-ethylmaleimide (dissolved in ethanol). Inhibitors were preincubated with the enzyme for 10 min at room temperature. The final concentration of the inhibitors was 1 mM in the assay. Control assays to check the effect of the solvents on activity were performed using the solvents in the absence of inhibitor.

### 2.8. Synthetic Peptide Plate Assays

Substrate specificity was determined using agarose plates containing synthetic peptides. A solution of 18.5 mL of 1.35% boiled agarose was cooled; then, synthetic peptide, DTT, MgCl_2_, and Mops buffer at pH 7.0 were added to give final concentrations of 0.65 mM synthetic peptide, 20 mM DTT, 10 mM MgCl_2_, and 50 mM Mops, in a total volume of 25 mL. The mixture was poured into a 100 × 15 mm plastic Petri dish. Wells were cut into the solidified agarose with a trimmed plastic dropper (diameter 4 mm). Samples of 28–40 *μ*L of PeiW (0.2 mg), PeiP (0.2 mg), or control buffer were pipetted into the wells and assay plates were incubated in a gas tight chamber under CO_2_ for 16 h at 39°C. The presence and size of a zone of yellow colouration were compared between different synthetic peptide substrates. The diameter of the zone could not be measured as the zone was diffuse and had no clearly defined edge.

### 2.9. Thermostability

Glycerol was removed from the PeiW preparation using a Bio-Gel P-6DG (Bio-Rad) column (2.5 × 17 cm) equilibrated with 20 mM Mops pH 7.0 containing 1 mM DTT and 0.3 M NaCl (as described above). Glycerol was removed from the PeiP preparation by repeated washing with 20 mM Mops pH 7.0 containing 1 mM DTT and 0.3 M NaCl in a 20 mL Vivaspin 10 kDa molecular weight cut-off concentrator (GE Healthcare, USA). Thermostability was measured in the presence and absence of 10 mM Ca^2+^. Aliquots of PeiW (3 mg mL^−1^) or PeiP (2.1 mg mL^−1^) were preincubated for 10 min at room temperature and then incubated at the desired temperature (70–85°C). At various intervals, aliquots were removed and cooled on ice. After mixing and centrifuging to remove any precipitate (14,100 g, 1 min, at room temperature), remaining activity was determined by assaying with E*γ*ApNA; 14 *μ*L PeiW or 75 *μ*L PeiP was assayed at 60°C, with assays being initiated by the addition of enzyme.

### 2.10. Molecular Mass Determination

The native molecular mass was determined by gel filtration chromatography. A standard curve was generated using aprotinin (6.5 kDa, Sigma-Aldrich, New Zealand), cytochrome c (12.4 kDa, Sigma-Aldrich, New Zealand), carbonic anhydrase (29 kDa, Sigma-Aldrich, New Zealand), bovine serum albumin (66 kDa, Sigma-Aldrich, New Zealand), alcohol dehydrogenase (150 kDa, Sigma-Aldrich, New Zealand), and *β*-amylase (200 kDa, Sigma-Aldrich, New Zealand). A filtered sample of each enzyme (400 *μ*L) at a concentration of 0.8 mg mL^−1^ was applied to a Superdex 200 (1 × 59 cm) column using a 1 mL loop. The column was eluted with 50 mM Mops pH 7.0 containing 1 mM DTT and 0.15 M NaCl at a flow rate of 0.6 mL min^−1^. The void volume of the column was determined using Blue Dextran (2,000 kDa, Sigma-Aldrich, New Zealand). The molecular mass was estimated from a standard curve prepared by plotting the elution volume versus the log of the molecular mass of the standards.

## 3. Results and Discussion

### 3.1. Synthetic Peptide Assay Development

A continuous assay measuring the release of* p*-nitroaniline (pNA) from synthetic peptides at 405 nm was developed for use in the characterisation of PeiW and PeiP. The synthetic peptides were designed based on the peptide chain of pseudomurein and incorporated pNA as a reporter molecule in place of lysine. The peptide portion of the synthetic peptides was sufficient to allow peptidase activity by PeiW and PeiP to cleave pNA. This assay was performed aerobically. Assays were performed at 60°C as the optimum growth temperature for* Mtb. wolfei* and* Mtb. marburgensis* is near 65°C and previous studies have used similar assay temperatures for PeiW and PeiP [8, 9, 14, 23]. As the extinction coefficient of pNA can vary depending on solution composition [30], the extinction coefficient of pNA was determined under assay conditions and found to be 10,140 M^−1^ cm^−1^. The synthetic peptides appeared to be stable under assay conditions, pH 7.85 and 60°C for up to 10 min, as no difference was found in initiating the reaction with enzyme or substrate.

**Figure 2:**
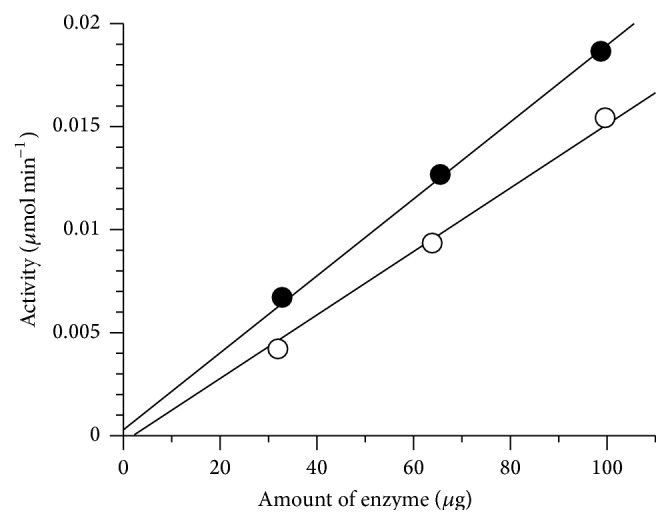
Linearity of synthetic peptide assay with amount of PeiW (●) or PeiP (○) using E*γ*ApNA as substrate.

The rate of activity in the synthetic peptide assay was proportional to the amount of PeiW or PeiP used, up to 100 *μ*g of enzyme per 800 *μ*L assay, with Glu-*γ*-Ala-pNA (E*γ*ApNA) as substrate (Figure 2).

The advantage of this continuous assay over a discontinuous assay is the ability to measure initial rates and therefore the ability to measure enzyme kinetics more easily. Additionally, methanogen cells and cell walls can be variable and are difficult and time consuming to produce and to date the assays have often been insensitive, anaerobic, discontinuous, and nonlinear [8]. Hence, there are limitations in their use for enzyme characterisation, including kinetic studies. Kiener et al. [8] showed nonlinearity with respect to the amount of PeiW crude extract in cell suspension assays. Morii and Koga [23] found that assaying with cell walls was linear with the amount of enzyme. The solution is the use of synthetic substrates, which are sensitive and well characterised, have high purity, and are easily obtained and their activity can be continuously measured spectrophotometrically.

The modified assay has a known extinction coefficient, pH, and calcium ion concentration and can be undertaken at 60°C. The assay is unable to be used with phosphate buffer, as the enzymes require the presence of a divalent metal. Phosphate buffer causes precipitation or binding of many divalent cations.

### 3.2. Purification and Characterisation

**Figure 3:**
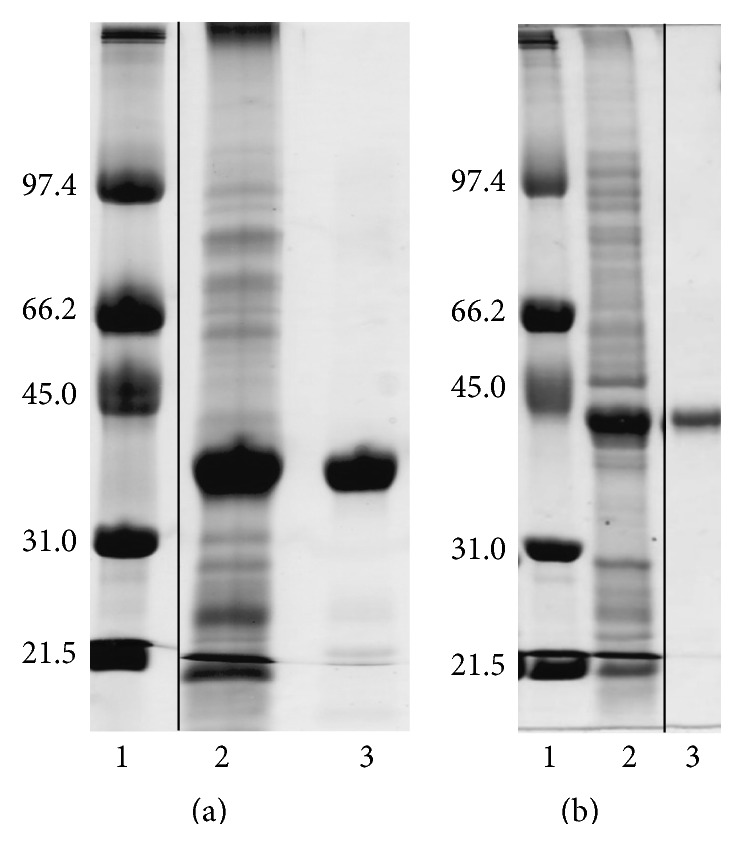
Purification samples of PeiW and PeiP on SDS-PAGE. Gel (a) PeiW; lane 1, molecular weight markers with sizes indicated in kDa, lane 2, PeiW crude lysate supernatant, and lane 3, PeiW after nickel-affinity chromatography step. Gel (b) PeiP; lane 1, molecular weight markers with sizes indicated in kDa, lane 2, PeiP crude lysate supernatant, and lane 3, PeiP after desalting step. A vertical line in each gel indicates the removal of irrelevant lanes.

Recombinant His-tagged PeiW and PeiP proteins were overexpressed in* E. coli* at high levels. The presence of an N-terminal His-tag allowed the enzymes to be purified to homogeneity in one step using nickel-affinity chromatography (Figure 3).

The apparent molecular mass of the purified recombinant PeiW was 26.8 kDa as determined by gel filtration chromatography (not shown) and 38 kDa as seen by SDS-PAGE, while that of PeiP was 32.3 kDa as determined by gel filtration chromatography (not shown) and 42 kDa as seen by SDS-PAGE. These values are close to those of 37,219 Da and 39,537 Da predicted for the His-tagged PeiW and PeiP proteins, respectively (317 and 338 amino acids), and indicate that PeiW and PeiP are both monomeric. The same result was obtained by Stax et al. [14] who also found PeiP to be monomeric using gel filtration chromatography.

**Figure 4:**
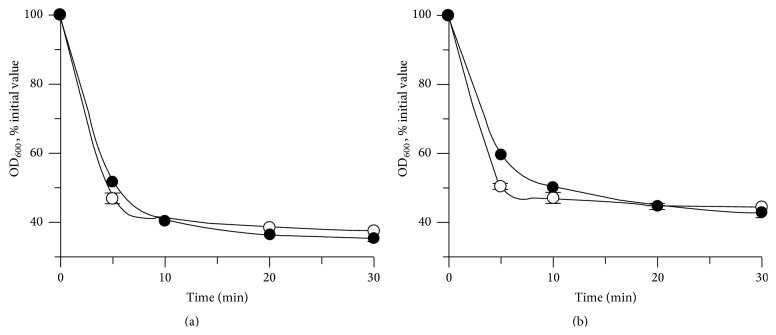
Activity of (a) PeiW and (b) PeiP in aerobic and anaerobic cell suspension assays. The assays were performed using* Mtb. thermautotrophicus* ΔH cells, as described in the Materials and Methods, ● aerobic, ○ anaerobic. Initial OD_600_ values were 0.771, PeiW aerobic; 0.865, PeiW anaerobic; 1.014, PeiP aerobic; 0.940, PeiP anaerobic. The total OD change of the enzyme-free and cell-free controls was less than 2% of the initial value.

While many studies have found that PeiW and PeiP have no activity when exposed to air, we found activity in both cell suspension and synthetic peptide assays under aerobic conditions in the presence of a reducing agent such as dithiothreitol (DTT) or tris(2-carboxyethyl) phosphine (TCEP). Cell suspension assay results were very similar whether performed aerobically or anaerobically (Figure 4). The OD shows a rapid decrease over the first 5 min but a very low decrease after 10 min. This may be due to clumped cell fragments and some precipitate, both only observable by microscope. This may be caused by released cellular components interacting with each other or with components in the assay. Anaerobic synthetic peptide assays showed similar results to cell suspension assays, with specific activities for PeiW of 0.12 ± 0.01 U mg^−1^ aerobically and 0.14 ± 0.02 U mg^−1^ anaerobically and specific activities for PeiP of 0.10 ± 0.01 U mg^−1^ aerobically and 0.10 ± 0.01 U mg^−1^ anaerobically. These results are in contrast to previous studies. Kiener et al. [8] found no activity of native PeiW if exposed to air for 1 hr at 37°C and culture assays required >30 mM potassium phosphate and reducing agents. Stax et al. [14] found that native PeiP had no activity if exposed to air in a culture assay. However, ~50% activity was recovered when anaerobic and reducing conditions were restored. Morii and Koga [23] found that native PeiW could be stored aerobically or anaerobically, but the highest activity was obtained when assayed anaerobically in the presence of DTT. When assayed aerobically, anaerobic storage resulted in much higher activity, as did the presence of DTT in the assay. It was proposed that oxygen inactivates and Na_2_S and DTT reactivate the enzyme. In this study, synthetic peptide assays were performed successfully aerobically which is likely due to reducing agents being present at all steps of enzyme manipulation from cell lysis to storage and assay.

**Figure 5:**
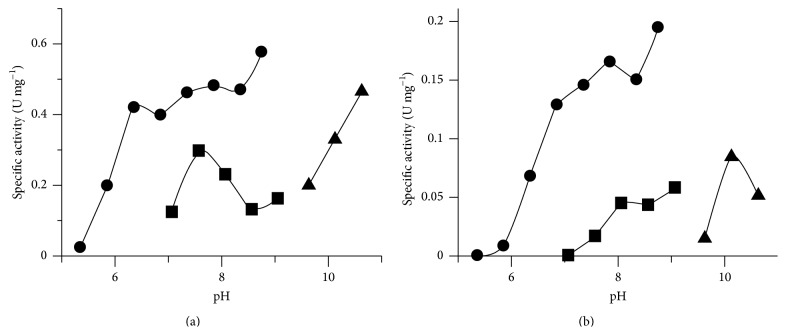
Effect of pH on activity of (a) PeiW and (b) PeiP in synthetic peptide assays. The assays were performed using standard conditions described in Section 2, buffers; ● Bistris propane, ■ Ampso, and ▲ Caps.

PeiW was active between pH 6.0 and pH 10.6 (Figure 5(a)). PeiP had a similar response as PeiW to pH (Figure 5(b)), with activity between pH 6.4 and pH 10.6. At comparable pH values, higher activity was found for both enzymes using 1,3-bis(tris(hydroxymethyl)methylamino)propane (Bistris propane) buffer compared to 3-[(1,1-dimethyl-2-hydroxyethyl)amino]-2-hydroxypropanesulfonic acid (Ampso). This was not the case for PeiW in the presence of Mg^2+^ where similar activities were seen at comparable pH values of Bistris propane buffer and Ampso (not shown). Ampso can form complexes with divalent metal ions [31, 32]. The lower activity using Ampso in the presence of Ca^2+^ may possibly be due to complex formation between Ca^2+^ and Ampso, while there may not be complexes formed to the same extent between Mg^2+^ and Ampso. A broad pH optimum has also been seen previously for native PeiP, with a pH optimum of 7.3 [14] using a culture assay. However, Morii and Koga [23] found the pH optimum of native PeiW to be between 6.8 and 7.4 using a cell wall assay. Luo et al. [9] reported the pH optimum for native PeiW, recombinant PeiW, and recombinant PeiP to be 6.4 using a resuspended cell assay.

**Figure 6:**
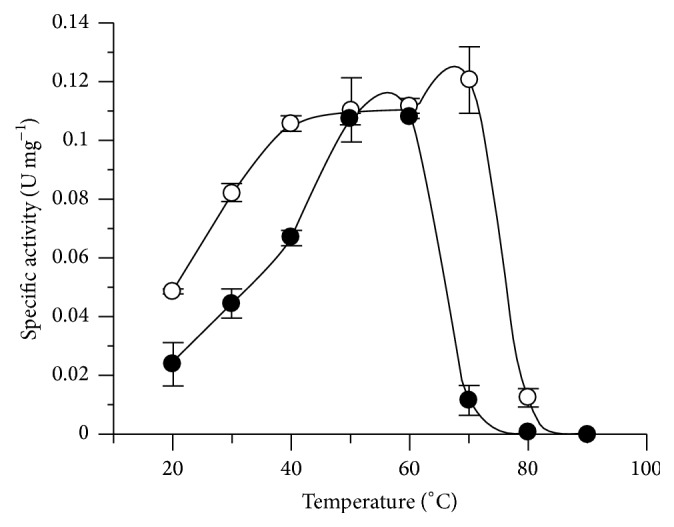
Effect of temperature on PeiW (○) and PeiP (●) using a synthetic peptide assay.

The effect of temperature on enzyme activity is shown in Figure 6. As the activity of PeiP decreased above 60°C, regular assays for both PeiW and PeiP were performed at 60°C. Kiener et al. [8] found that native PeiW was not active at 37°C, but activity increased over the temperatures 45, 52, and 62°C in a culture assay. Native PeiP has previously been reported to have a maximal activity at 62°C, in a culture assay [14]. Morii and Koga [23] found maximal activity of native PeiW at 75°C in cell wall assays. Luo et al. [9] reported maximal activity of native PeiW at 67°C, recombinant PeiW at 71°C, and recombinant PeiP at 63°C, as determined using resuspended cell assays.

**Figure 7:**
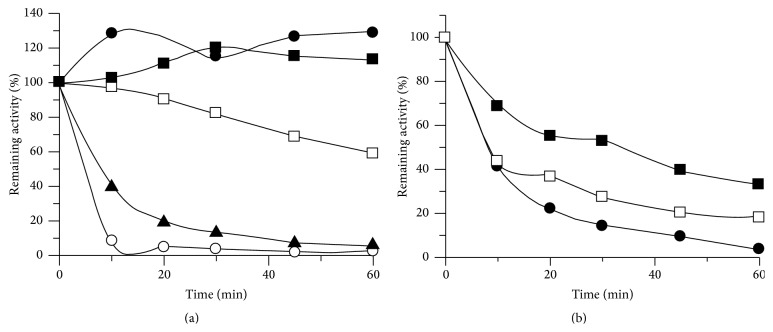
Thermostability of (a) PeiW and (b) PeiP in the presence and absence of Ca^2+^. (a) Thermostability of PeiW, in the presence of Ca^2+^, ■ 75°C, ● 80°C, and ▲ 85°C, and in the absence of Ca^2+^, □ 75°C, ○ 80°C. (b) Thermostability of PeiP, in the presence of Ca^2+^, ■ 70°C, ● 75°C, and in the absence of Ca^2+^, □ 70°C. Initial specific activity values (U mg^−1^) were 0.253, PeiW and 0.0429, PeiP.

Both PeiW and PeiP showed much greater thermostability in the presence of 10 mM Ca^2+^. For PeiW at 80°C, when Ca^2+^ was present, 100% of the initial activity remained after 60 min; however, in the absence of Ca^2+^, 50% of activity remained after only 5 min (Figure 7(a)). A similar effect was observed with PeiP but at a lower temperature; at 70°C, when Ca^2+^ was present, 50% of the initial activity remained after 30 min, but in the absence of Ca^2+^, 50% of activity remained after only 8 min (Figure 7(b)). Both enzymes were thermostable at 60°C, the temperature used for regular assays. Inclusion of cell walls has previously been found to improve thermostability. Morii and Koga [23] reported that native PeiW incubated at 75°C was stable for some time in the presence of cell walls, while 50% of activity was lost after 10 min with no cell walls. Native PeiW incubated at 100°C for 10 min displayed no activity, but activity was restored by prolonged incubation with cell walls at 62°C (18 h). No activity was restored if native PeiW was incubated without cell walls and one-hour incubation at 100°C gave complete inactivation. Morii and Koga [23] used cell wall assays to determine the remaining activity. The results for PeiW and PeiP presented here are consistent with the optimum growth temperature for* Mtb. wolfei* and* Mtb. marburgensis* of near 65°C. The results are also consistent with the thermostability exhibited by other proteins isolated from thermophilic organisms.

The activity of the enzymes treated with 0.5 mM EDTA and assayed in the absence of metal ions was less than 1% of the untreated enzymes when assayed in the presence of Ca^2+^. When assayed with Ca^2+^ present, activities of the EDTA-treated enzymes were the same as those of the untreated enzymes. These results indicate that a metal ion is required for catalytic activity. A range of divalent metal salts was tested for their ability to restore activity to the EDTA-treated PeiW and PeiP (Table 1). PeiW was activated by Ca^2+^, Mn^2+^, Mg^2+^, Ba^2+^, and Ni^2+^ in that order. However, PeiP was significantly activated by Ca^2+^ only. Mn^2+^, Mg^2+^, Ba^2+^, and Ni^2+^ resulted in less than 15% of the activity with Ca^2+^. Luo et al. [9] also found that EDTA inhibited activity of recombinant PeiW and PeiP, which could be recovered by the addition of Ca^2+^ or Mg^2+^, as assayed using resuspended cells.

**Figure 8:**
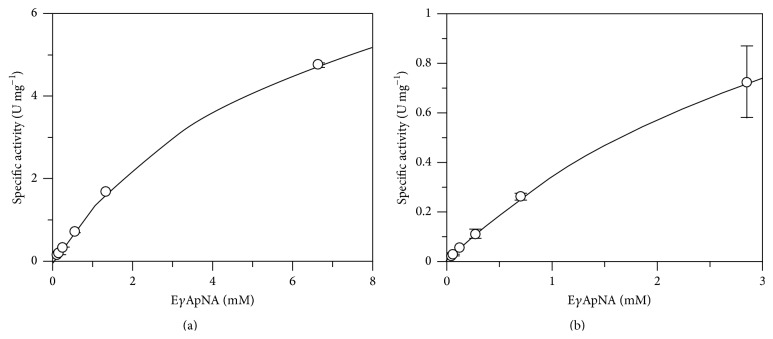
Michaelis-Menten plots for (a) PeiW and (b) PeiP, in synthetic peptide assays.

PeiW and PeiP appeared to follow Michaelis-Menten kinetics using the substrate E*γ*ApNA (Figure 8). The apparent kinetic constants for PeiW were *K*_*M*_  6.25 ± 0.44 mM and *k*_cat_  5.73 ± 0.22 s^−1^, and those for PeiP were *K*_*M*_  4.14 ± 0.14 mM and *k*_cat_  1.18 ± 0.03 s^−1^, at 60°C and pH 7.85. Due to the high *K*_*M*_ values and the limited solubility of E*γ*ApNA, the range of substrate concentrations in the kinetics experiments was limited to 1 × *K*_*M*_ or less. The determined *K*_*M*_ values are in the mM range indicating that PeiW and PeiP have low affinities for E*γ*ApNA. While this synthetic substrate reflects some aspects of the natural substrate, it is not as large and this may lead to poorer binding. It is unlikely that the synthetic peptides are large enough to be bound to the pseudomurein cell wall binding domain at the N-termini of PeiW and PeiP [10, 18]. Additionally, the bond cleaved in the natural substrate is the Ala-*ε*-Lys bond while Ala-pNA is cleaved in the synthetic substrate, which could also be less favourable.

The inhibitors tested were known peptidase inhibitors.* N*-Ethylmaleimide, a cysteine protease inhibitor, inhibited PeiW (80% inhibition) and PeiP (90% inhibition) at 1 mM concentration. Cystamine, a cysteine protease inhibitor, showed little inhibition unless TCEP was absent in the assay, in which case there was 100% inhibition. In the presence of TCEP, cystamine would be reduced, rendering it inactive as a peptidase inhibitor. There was poor inhibition by E64 (a cysteine protease inhibitor) and PMSF (a serine protease inhibitor) of PeiW (10% and 10% inhibition, resp.) and PeiP (30% and 20% inhibition, resp.). Dansylcadaverine, a transglutaminase inhibitor, inhibited PeiW (60% inhibition) and PeiP (50% inhibition) at 1 mM concentration. Cysteine has been identified in the conserved catalytic triad of transglutaminases and thiol proteases shared by PeiW and PeiP [9, 33]. The inhibition by cystamine and* N*-ethylmaleimide confirms that a cysteine residue is important in PeiW and PeiP catalysis. Inhibition by dansylcadaverine supports PeiW and PeiP having the same catalytic triad as that of transglutaminases.

Substrate specificity was determined on a variety of synthetic peptide substrates (Table 2) using an agarose plate activity assay. This type of assay was used as activity on most synthetic peptides was too low to be reliably determined over a short time period in the continuous assay. The agarose plate assay was anaerobically incubated overnight to give the highest chance of detecting activity. Both PeiW and PeiP were most active on E*γ*ApNA, which was used for the characterisation of PeiW and PeiP. This is consistent with lysis of the *ε*-isopeptide bond between alanine and lysine (Figure 1) by these enzymes in pseudomurein, as shown by Stax et al. [14]. Both enzymes showed some activity on H-Glu-Ala-pNA (EApNA). This indicates that the peptide bond between glutamate and alanine is not strictly required to be a *γ*-isopeptide bond for activity to occur. l-Ala-pNA (ApNA) was a poor substrate compared to EApNA or E*γ*ApNA, indicating that these latter larger molecules had a much better ability to bind to the active site of PeiW and PeiP and that these molecules have a more similar structure to the native substrate of the enzymes, pseudomurein. ApNA may have been too small to bind effectively. Asp-*β*-Ala-pNA (D*β*ApNA) is a very similar molecule to E*γ*ApNA but was a poor substrate, signifying that aspartate cannot substitute for glutamate and that the native substrate of PeiW and PeiP is unlikely to contain aspartate in this position. Activity on Glu-*γ*-Ser-pNA (E*γ*SpNA) shows that alanine is not strictly needed as the very similar amino acid serine can substitute, despite serine not being present in the pseudomurein of* Mtb. marburgensis* or* Mtb. wolfei*, the native substrates of PeiP and PeiW, respectively. However, serine is found in this position in the pseudomurein of some methanogens [3, 7]. Threonine appears unable to substitute for alanine, as we could detect no activity using Glu-*γ*-Thr-pNA (E*γ*TpNA). This is consistent with threonine not being found in the pseudomurein of* Mtb. marburgensis*, the native substrate of PeiP [3]. Our results suggest that threonine is also unlikely to be found in the pseudomurein of* Mtb. wolfei*, the native substrate of PeiW.

We also determined the substrate specificity toward a variety of methanogen cells, using plate lysate assays (Table 3). A zone of clearing was observed which sometimes included an inner zone of whitening. The whitening may have been due to released cellular components interacting with each other or with components in the plate. PeiW and PeiP lyse* Mbb. gottschalkii*,* Mbb. smithii*,* Mbb. ruminantium* strain 31A,* Msph. stadtmanae*,* Mb. formicicum* strain BRM9, and* Mtb. thermautotrophicus* ΔH. Additionally, PeiW lyses* Mbb.* sp. SM9. PeiW and PeiP did not lyse* Mbb. ruminantium* strain M1,* Msp. hungatei*, and* Ms. barkeri* strain CM1. The latter two methanogens were included as controls as they do not contain pseudomurein in their cell wall; rather, they contain glycoprotein and methanochondroitin, respectively [1, 3]. The lysis of* Msph. stadtmanae* cells and the nonlysis of* Mbb. ruminantium* strain M1 are consistent with the results for synthetic peptides, as* Msph. stadtmanae* pseudomurein may contain serine in place of alanine in its pseudomurein, while* Mbb. ruminantium* strain M1 pseudomurein may contain threonine in place of alanine [3, 7]. Our results are in general agreement with those of others. PeiW and PeiP were found to lyse cell suspensions of* Mtb. marburgensis* [9, 13]. Morii and Koga [23] tested PeiW against methanogen cell suspensions and found slow lysis of* Mbb. arboriphilus* (DH1, AZ, DC, and A2) but no lysis of* Msph. stadtmanae*. König et al. [34] tested* Mtb. wolfei* lysates against methanogen cell suspensions finding that* Mtb. wolfei*,* Mtb. marburgensis*,* Mtb. thermautotrophicus* (ΔH),* Mb. bryantii* MoH, and* Mb. formicicum* were lysed, while* Mb. bryantii* MoHG,* Mbb. smithii*,* Mbb. arboriphilus*,* Msp. hungatei*,* Methanogenium tatii*, and* Methanothermus fervidus* were not. However,* Mth. fervidus* cells were reported to be lysed by PeiW by Kiener et al. [8].* Msp. hungatei* and* M. tatii* are not expected to be lysed as they do not possess pseudomurein [3, 7]. Any differences between results are likely to be due to different methods of assay, different strains of a species, or different procedures of preparing methanogen cells.

## 4. Conclusions

We have developed a continuous synthetic peptide assay which could be used at elevated temperatures and have used this in the biochemical characterisation of recombinant PeiW and PeiP. The series of synthetic peptides that we have developed mirrors the bonds within the peptide chain of pseudomurein. The assay solves the problems associated with the previously used natural substrates which are variable, difficult, and time consuming to produce and result in assays which are often anaerobic, discontinuous, and nonlinear. Synthetic substrates are sensitive and well characterised, have high purity, and are more easily obtained and activity is continuously measured spectrophotometrically. Additionally, assays were performed aerobically in this study. The synthetic peptide assay has allowed us to characterise PeiW and PeiP more fully and obtain more reliable and reproducible results. The results from these substrates are consistent with lysis of the *ε*-isopeptide bond between alanine and lysine by these enzymes in pseudomurein. Information about the specific structural variations of the particular native pseudomurein substrate of these enzymes has also been inferred. The enzymes were shown to require a divalent metal and to involve a cysteine residue in catalysis. This new assay may have wider applications for both the general study of peptidases and the identification of which methanogens are susceptible to lysis by specific pseudomurein endoisopeptidases.

## Figures and Tables

**Table 1:** Activation (% activity) of PeiW or PeiP by divalent metal ions in a synthetic peptide assay. 100% activity corresponded to 0.245 U mg^−1^ for PeiW and 0.115 U mg^−1^ for PeiP.

Metal ion present	% activity	
	PeiW	PeiP
Ca^2+^	100	100
Mn^2+^	78	8
Mg^2+^	66	14
Ba^2+^	51	12
Ni^2+^	42	3
Co^2+^	0	0
Cu^2+^	0	0
EDTA	0	0

**Table 2:** Substrate specificity, activity of PeiW or PeiP on synthetic peptide substrates using an agarose plate assay. The intensity of a zone of yellow colouration was compared; − no zone, + zone being only just observable, and +++++ very large zone. No yellow zones were observed with control buffer in the wells.

Synthetic peptide substrate	Abbreviation	Relative activity	
		PeiW	PeiP
L-Ala-pNA	ApNA	+	+
H-Glu-Ala-pNA	EApNA	+++	++
Glu-*γ*-Ala-pNA (Glu(Ala-pNA)-OH)	E*γ*ApNA	+++++	+++++
Glu-*γ*-Ser-pNA (Glu(Ser-pNA)-OH)	E*γ*SpNA	+++	++
Glu-*γ*-Thr-pNA (Glu(Thr-pNA)-OH)	E*γ*TpNA	−	−
Asp-*β*-Ala-pNA (Asp(Ala-pNA)-OH)	D*β*ApNA	+	+
L-Glu-*γ*-pNA (Glu(pNA)-OH)	E*γ*pNA	+	−

**Table 3:** Substrate specificity, activity of PeiW or PeiP on methanogen cells as substrates using an agarose plate lysate assay. Activity is diameter (mm) of clear zone produced in agarose plate. No clear zone is indicated by −. The diameter of the well was 4 mm. No clear zones were observed with control buffer in the wells.

Methanogen cell substrate	Activity, diameter of clear zone (mm)	
	PeiW	PeiP
*Methanobacterium formicicum* strain BRM9	22	18
*Methanosphaera stadtmanae *(DSM 3091)	19	20
*Methanothermobacter thermautotrophicus* ΔH	19	18
*Methanobrevibacter ruminantium* strain 31A	21	16
*Methanobrevibacter smithii* (DSM 861)	20	17
*Methanobrevibacter gottschalkii *(DSM 11977)	20	12
*Methanobrevibacter *sp. SM9	10	−
*Methanobrevibacter ruminantium* strain M1	−	−
*Methanosarcina barkeri* strain CM1	−	−
*Methanospirillum hungatei *(DSM 864)	−	−
No cells (buffer control)	−	−

## Abbreviations

Ampso: 3-[(1,1-Dimethyl-2-hydroxyethyl)amino]-2-hydroxypropanesulfonic acid
ApNA: l-Ala-pNA
Bistris propane: 1,3-Bis(tris(hydroxymethyl)methylamino)propane
Caps: 3-(Cyclohexylamino)-1-propanesulfonic acid
D*β*ApNA: Asp-*β*-Ala-pNA
DTT: Dithiothreitol
EApNA: H-Glu-Ala-pNA
E*γ*ApNA: Glu-*γ*-Ala-pNA
E*γ*pNA: l-Glu-*γ*-pNA
E*γ*SpNA: Glu-*γ*-Ser-pNA
E*γ*TpNA: Glu-*γ*-Thr-pNA
FISH: Fluorescence* in situ* hybridisation
PEG: Polyethylene glycol
Pei: Pseudomurein endoisopeptidase
pNA: * p*-Nitroaniline
TCEP: Tris(2-carboxyethyl) phosphine.
